# Weight, body composition and quality of life changes in a Hungarian community-based body weight management program: an observational cohort analysis

**DOI:** 10.3389/fpubh.2026.1751402

**Published:** 2026-02-19

**Authors:** Éva Csajbók, Sándor Bordé, Terézia Páhi, Anita Kollárné Korsós, Zsuzsanna Gyurisné Pethő, Anna Vágvölgyi, Árpád Kallai

**Affiliations:** 1 Hódmezővásárhely - Makó Healthcare Centre, Hódmezővásárhely and Makó, Hungary; 2 Endocrinology and Diabetology Center, Department of Medicine, Albert Szent-Györgyi Medical School, University of Szeged, Szeged, Hungary; 3 MTA-SZTE Research Group for Cortical Microcircuits, Department of Anatomy, Physiology and Neuroscience, University of Szeged, Szeged, Hungary

**Keywords:** body composition, body weight management program, obesity, overweight, quality of life

## Abstract

**Introduction:**

Overweight and obesity represent major global public health challenges. We designed a prevention-oriented lifestyle program targeting adults living with overweight or mild obesity, with the primary objective of preventing further weight gain and improving overall health through a multidomain intervention in a real-world community setting.

**Methods:**

We retrospectively analyzed data from a non-randomized, prospective observational study. Adults aged 18–65 years (BMI 25–35 kg/m^2^) motivated to improve their quality of life participated in a 9-month community-based educational program (“Body Weight Management Program”) in a Hungarian township (2015–2022). The intervention consisted of regular medical, nutritional, and psychological consultations, complemented by compulsory individualized physical training. Clinical parameters were assessed at baseline (month 0) and reevaluated at 3, 6, and 9 months, and at 21 and 33 months post-intervention. Quality of life was assessed using the ORWELL-97 questionnaire at baseline, at the end of the active program, and at 21 and 33 months.

**Results:**

Three hundred eight subjects were enrolled (281 women, 27 men; data are presented as mean and quartiles [Q1; Q3]: BMI: 30.5 kg/m^2^ [28.1; 32.3]). Participant retention declined over time, with 88% at 3-months visit, 67% at 6-months visit, and 68% completing the 9-month program. Long-term follow-up data were available for only a subset of participants. BMI decreased gradually during the intervention with a mean absolute change of −1.57 kg/m^2^ (95% CI: −1.81 to −1.32) by the end of the program. The corresponding mean absolute body weight reduction was −4.30 kg (95% CI: −4.97 to −3.64), body fat percentage decreased by −3.02 percentage (95% CI: −3.62 to −2.41). Quality of life improved significantly and durably, with the OxR score decreasing by a mean of 76.06 points (95% CI, −89.33 to −62.79) from baseline to the end of the intervention, and the improvement was sustained at 21- and 33-month follow-up.

**Conclusion:**

Participation in our newly developed nine-month program was associated with a marked and durable improvement in quality of life, which was maintained up to 33 months of follow-up. Although the observational design precludes causal inference, the persistence of these improvements suggests a potentially meaningful long-term benefit.

## Introduction

Obesity is defined as a chronic and recurring disease caused by abnormal or excessive fat accumulation. According to the body mass index (BMI), overweight (OW) is defined as a BMI of 25–29.9 kg/m^2^, while obesity (OB) is defined as a BMI of ≥30 kg/m^2^ (1). Besides BMI, body composition is an important contributing factor to consequences such as cardiovascular diseases (2), type 2 diabetes mellitus, stroke (3), dementia (4), obstructive sleep apnea (5), and several cancers (6, 7), all of which lead to a decline in quality of life (8) and life expectancy (9). Obesity is also associated with social disadvantages (10), reduced productivity (11), and higher healthcare costs (12), creating an economic burden. Therefore, obesity prevention – both at the individual and population levels – should be promoted (13). The prevalence of OB has increased worldwide in the past decades, reaching pandemic levels. Hungary is among the leading countries with a high prevalence of OW and OB, contributing to high cardiovascular and cancer mortality rates. According to the latest data from the World Obesity Global Obesity Observatory, 34.4% of the Hungarian adult population is overweight, and 23.8% is obese (men: OW: 40.7%, OB: 24.6%, women: OW: 32.1%, OB: 20.0%) (14). A Hungarian study group performed an analysis using linked questions from the 2009, 2014, and 2019 European Health Interview Survey databases, which are considered representative of the Hungarian adult population. According to their data, the prevalence of OW and OB was 56% in 2009 and 55% in 2014; by 2019, this figure had increased to 62%. By gender, 64% of men and 52% of women were overweight or obese (15).

Although behavioral interventions that reduce caloric intake and increase energy expenditure can achieve short-term weight loss, their long-term effectiveness is limited by complex and persistent hormonal, metabolic, and neurochemical adaptations that defend against weight reduction and promote weight regain. Nonetheless, increasing individuals’ understanding of the physiological and pathophysiological mechanisms underlying obesity remains crucial for promoting engagement in treatment and long-term self-management. Reducing the population-level burden of obesity requires strategies that integrate individual-level interventions with broader environmental and societal changes (16).

U. S. Preventive Services Task Force recommends routine obesity screening in primary care and the provision of intensive, multicomponent behavioral interventions for adults when indicated (17). The 2013 Guidelines for Managing Overweight and Obesity emphasize high-intensity lifestyle programs of at least 6 months’ duration, including at least 14 counseling sessions delivered by trained professionals. Evidence suggests that reduced caloric intake, regular physical activity, and frequent self-monitoring of body weight (BW) are associated with sustained weight loss, while maintenance programs that provide at least monthly counseling during the first year are critical for preventing weight regain. To support long-term weight management, efforts have increasingly focused on expanding access to lifestyle modification through community-based programs (18).

Large randomized controlled trials, such as the Diabetes Prevention Program (DPP) (19), Look AHEAD (Action for Health in Diabetes) (20), Da Qing Diabetes Prevention Study (21), and DiRECT (Diabetes Remission Clinical Trial) (22) studies, have clearly demonstrated that intensive, multidisciplinary lifestyle interventions can achieve clinically meaningful weight loss and improve metabolic and quality-of-life outcomes. While these trials provide strong evidence for the efficacy of structured lifestyle modification under controlled conditions, their findings may not be directly transferable to routine public health practice. Participants in such trials are typically highly selected, interventions are resource-intensive, and implementation occurs within well-controlled research environments. Consequently, real-world evidence from community-based settings—particularly in Central and Eastern European countries—is limited.

In 2008, the Municipality of Hódmezővásárhely in southern Hungary launched a public health initiative [*Healthy Hódmezővásárhely Program (EVP), Egészséges Vásárhely* Program] (23) with the mission to improve public health, support disease prevention, and encourage healthy lifestyle habits. At that time, there was no unified, nationwide, publicly funded lifestyle-based weight management program available in Hungary. In 2015, recognizing a strong need to reduce the obesity burden in the region the EVP requested a multidisciplinary team to design and implement an educational, complex program for people living with overweight and mild obesity, with the aim of enhancing health literacy and promoting sustainable lifestyle changes to prevent weight gain and reduce the risk of progression to severe obesity [*Body Weight Management Program (BWMP), Testsúlykontroll Program*]. The primary objective of the BWMP was to improve the overall health status, quality of life, physical and mental fitness of individuals living with excessive body fatness, aiming to prevent the metabolic, cardiovascular, and psychosocial consequences as well as to empower participants to disseminate health-promoting practices within their families and local communities. Participants who volunteered for health assessments organized by the EVP in two cities (Makó and Hódmezővásárhely) were referred to the BWMP if they met the eligibility criteria.

## Methods

### Ethical approval

The retrospective analysis of prospectively collected data was approved by the Regional Medical Research Ethics Committee of the Albert Szent-Györgyi Clinical Center, University of Szeged (approval No. 267/2019). All individuals who entered the BWMP signed a formal participation agreement with the Hódmezővásárhely - Makó Healthcare Centre. This study was carried out in accordance with the Declaration of Helsinki (revised in 2013) of the World Medical Association.

### Study design and setting of BWMP

This study was a retrospective analysis of prospectively collected data from a real-world, municipality-run, community-based program, without randomization and without a control group. The BWMP was developed as a multidisciplinary intervention for adults living with overweight and mild obesity in a small community in southern Hungary, with participant recruitment conducted between October 2015 and December 2022. Because this was a retrospective analysis of a real-world community program, the study was neither registered nor preregistered. The program consisted of a 9-month educational lifestyle intervention, followed by assessments at 1 and 2 years post-completion. Baseline evaluations and follow-up measurements were conducted at two clinical sites. This study was conducted and reported in accordance with the Strengthening the Reporting of Observational Studies in Epidemiology (STROBE) guidelines for cohort studies (Supplementary Table 1) STROBE checklist (24).

### The aim and structure of the BWMP, recruitment data

Our program was designed to train, educate, and support participants in incorporating healthier eating habits, better food choices, conscious energy balance management, and regular physical activity into their daily lives, with the help of medical, physical, and psychological support. With comprehensive medical and psychological support, the program aimed to prevent or delay the potential complications associated with OW and OB.

**Figure 1:**
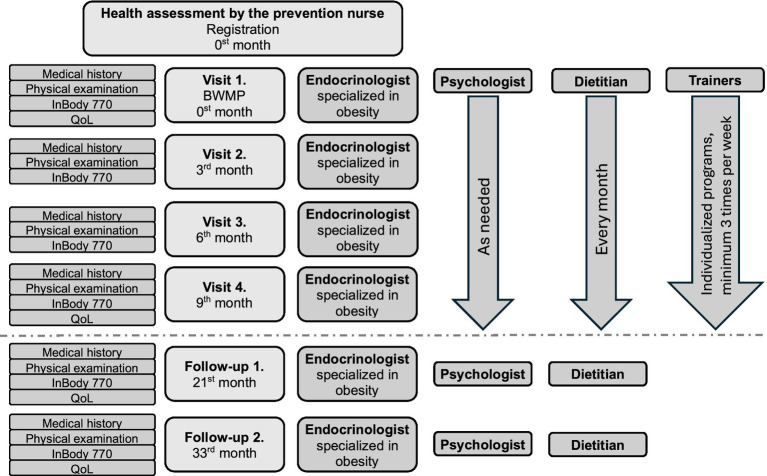
Structure and scheduled visits of the Body Weight Management Program and follow-up period. BWMP, Body Weight Management Program; QoL, quality of life questionnaire. The dashed line illustrates the end of the program and the beginning of the follow-up.

The EVP provides a wide range of services to support residents of the Hódmezővásárhely Microregion (19). At the EVP Health Promotion Office, individuals could apply to have their health status assessed. The initial consultation was conducted by a prevention nurse, who completed a risk assessment and, based on this, referred the applicant to the appropriate program (e.g., smokers to the smoking cessation program). If the prevention nurse classified the applicant as overweight or mildly obese based on BMI, participation in the BWMP was offered (see Registration, Figure 1).

The applicant was then scheduled for a baseline consultation (Visit 1, Month 0), during which detailed information about the program and its requirements was provided. This visit included a comprehensive medical history and an internal medicine–focused clinical examination. If the applicant was deemed to participate, they were given a participation agreement, which they were required to sign. By doing so, they agreed to attend the scheduled medical and dietary consultations and to participate in the individually prescribed physical activity sessions. Participation was voluntary, free of charge irrespective of the number of consultations or training sessions, and participants were free to withdraw at any time. During the active phase of the program, medical consultations were conducted every 3 months, dietary consultations were held monthly, and psychological consultations took place at the first and final visits and as needed. In addition, participation in structured, individualized exercise programs, supervised by certified trainers at least three times per week, with a minimum duration of 50 min per session, was mandatory (Figure 1). The managing team reserved the right to discontinue participation in cases of insufficient compliance. During the active phase and throughout the 2-year follow-up period, participants were allowed to access additional medical, dietary, and psychological consultations as needed; however, free supervised training programs were no longer provided during follow-up. Although the program was provided at no cost, participants were informed of its approximate value and were advised not to use meal replacements or pharmacological weight-management therapies during participation.

### Inclusion criteria

Participants were eligible for inclusion if they met all of the following criteria:

1. Age between 18 and 65 years.
2. BMI between 25 and 35 kg/m^2^.
3. Demonstrated motivation to adopt and maintain a healthier lifestyle, assessed during the baseline consultation.
4. Ability and willingness to participate in a structured lifestyle intervention program and attend scheduled follow-up visits.
5. Provision of written participation agreement after receiving a detailed explanation of the program.
6. Presence of hypertension and/or hypothyroidism was permitted, provided that the condition was clinically stable and adequately controlled with medication prior to enrollment.

### Exclusion criteria

Participants were excluded if any of the following criteria were present at baseline:

1. Diagnosis of type 1 or type 2 diabetes mellitus.
2. Active malignant disease.
3. Major obesity-related organ damage, including but not limited to:
  - Recent myocardial infarction,
  - Advanced heart failure,
  - Severe diabetes-related complications,
  - Disabling osteoarthritis limiting physical activity,
  - Child–Pugh class B or C liver cirrhosis,
  - impaired renal function.
4. Severe psychiatric disorders (e.g., schizophrenia, psychotic or suicidal depression, bipolar disorder).
5. Alcohol or drug dependence.
6. Significant cognitive impairment interfering with informed consent or adherence.
7. Diagnosed eating disorders.
8. Pregnancy at baseline.
9. Use of anti-obesity agents (pharmacological treatments, meal-replacement formulas, or herbal preparations).

Pregnancies occurring during the intervention resulted in temporary suspension of participation until postpartum reassessment.

Participants were withdrawn from the program if they repeatedly failed to attend scheduled visits, or showed persistent non-adherence to the prescribed physical activity protocol.

### Medical consultation, clinical variables, body composition

At baseline, all participants underwent a standardized clinical assessment performed by an endocrinologist specializing in obesity medicine. Anthropometric measurements were collected, and dietary habits and physical activity patterns were assessed using structured questionnaires. Medical and family history, current pharmacotherapy, and relevant laboratory parameters were reviewed and documented. Eligibility was confirmed based on predefined inclusion and exclusion criteria. Individualized daily energy intake targets for the 9-month intervention period were then calculated to achieve the pre-specified BW reduction goal.

Physical examinations were conducted at baseline and during scheduled follow-up visits at 3, 6, and 9 months, with additional assessments performed upon request by participants. Clinical variables, including waist circumference (WC), hip circumference (HC), blood pressure (BP), and pulse rate (P), were recorded. These variables were reassessed at the 3-, 6-, and 9-month follow-up visits during the intervention phase, and again at 1 and 2 years post-intervention to assess long-term outcomes.

At the initiation of the program, financial limitations restricted body composition assessment to the use of the OMRON BF511 body scale and body composition monitor between October 2015 and August 2018. From August 2018 to December 2022, measurements were performed using an InBody770 analyzer, which provided higher-resolution, multi-parameter body composition data. Body composition analysis was performed at baseline and at each subsequent visit.

### Dietary intervention

Participants were instructed to complete a 2-week dietary log to estimate their mean daily energy intake. Throughout the 9-month program, they attended monthly dietary consultations conducted by a certified dietitian. Additional consultations were available upon request. At each visit, body composition was measured, and individual energy requirements were recalculated to support sustained weight reduction. The dietitian determined individually daily energy targets and provided guidance on reducing energy intake and modifying dietary patterns. Macronutrient balance was optimized using the Smart Plate (OKOSTÁNYÉR^®^) (25) dietary framework.

### Physical activity

A certified training advisor provided detailed information regarding the available physical activity options and recommended individualized activity combinations tailored to participants’ clinical status and physical capacity. Personalized training programs were developed and supervised by certified trainers. Participants were required to engage in a minimum of three training sessions per week, each lasting at least 50 min, including at least two cardiovascular sessions. Home-based exercise modalities (e.g., walking, running, cycling, and swimming) were permitted without restriction; however, participants received guidance on recommended exercise types and intensities appropriate for their cardiovascular condition. The program offered a wide range of supervised activities, including Nordic walking, swimming, yoga, Pilates, Zumba, running, spinning, Jiu-Jitsu, Total Resistance eXercise (TRX), Natural Movement Pattern Training (NMPT), and various indoor interval and endurance training sessions. Adherence to the minimum prescribed weekly physical activity was mandatory for continued participation in the program, and failure to meet this criterion resulted in discontinuation from the intervention.

### Psychological support

Psychologist consultations were conducted at baseline and at the completion of the active intervention phase. Additional individual sessions could be scheduled upon request, and in some cases were provided more frequently to support behavioral change and the adoption of healthier lifestyle patterns. Group-based psychological sessions were also organized to enhance and sustain participant motivation. Standardized psychological instruments were administered, including assessments of eating behavior, self-esteem, and stress management. Health-related quality of life was evaluated using the ORWELL-97 (26), administered at baseline, at the 9-month visit, and at 1- and 2-year follow-up assessments by either the endocrinologist or the psychologist.

### Biometric, anthropometric data

The WC was measured at the midpoint between the lower margin of the last palpable rib and the upper border of the iliac crest. HC was measured at the level of the widest part of the buttocks. The waist-to-hip ratio (WHR) was calculated by dividing waist circumference by hip circumference (WHR = WC/HC).

Blood pressure was assessed using an OMRON Blood Pressure Monitor M3 on the left brachial artery, with participants in a seated position. Measurements were taken after 10-min rest period to minimize the risk of ‘white-coat hypertension’. Systolic (SBP, mmHg) and diastolic (DBP, mmHg) blood pressure, as well as pulse rate (P, beats per minute), were documented at each visit.

### Body composition analysis

At the beginning of the program, body composition analysis was conducted using the OMRON BF511 Body Composition and Body Fat Monitor (OMRON Corporation, Shiokoji Horikawa, Shimogyo-ku, Kyoto 600–8,530, Japan). This device calculated and documented BMI (kg/m^2^), body fat percentage (BF, %), muscle mass percentage (MM, %), visceral fat percentage (VF, %), and basal metabolic rate (BMR, J).

From August 2018 to December 2022, body composition was assessed using the InBody 770 segmental bioimpedance analyzer (InBodyUSA, Cerritos, CA). The device provided measurements -among others not included in the present analysis – of BW, BMI, total body fat mass (BFM) and percentage body fat (BFP), skeletal muscle mass (SMM), fat-free mass (FFM), fat-free mass index (FFMI), bone mineral content (BMC), basal metabolic rate (BMR), visceral fat area (VFA), and the InBody Score (IBS).

All measurements were performed according to standardized protocols: participants were lightly clothed, barefoot, and measured in a fasted state (≥3 h postprandial) at similar times of day. Measurements were conducted by trained staff to minimize inter-observer variability.

To account for device heterogeneity, we performed a sensitivity analysis restricted to measurements obtained with the InBody 770 (2018–2022). While the reduced sample size (approximately 50%) led to a slight increase of the *p*-values due to the loss of statistical power, the results remained consistent with the full dataset. This suggests that the introduction of the second device did not materially affect the study outcomes.

### Obesity related well-being questionnaire: ORWELL-97

Quality of life was assessed using the ORWELL-97 questionnaire (26), which evaluates the intensity and perceived relevance of physical and psychosocial distress related to overweight and obesity. Total scores are calculated by multiplying occurrence (O) and relevance (R) scores for each item and summing the resulting products (O × R). Higher scores indicate greater distress and poorer overall quality of life.

### Rationale and implications for generalizability

The inclusion criteria were designed to enroll adults with overweight or class I obesity who were medically stable and capable of safely participating in a lifestyle intervention, thereby minimizing confounding by severe comorbidities and reducing the risk of adverse events. Exclusion of individuals with diabetes, advanced organ damage, or severe psychiatric conditions aimed to ensure participant safety and intervention feasibility but may limit the generalizability of the findings to metabolically healthier overweight and mildly obese populations. Consequently, results should be interpreted primarily in the context of adults with overweight or class I obesity without advanced obesity-related complications.

### Statistical analysis

Normality was assessed using the Shapiro–Wilk test, which indicated that majority of the variables showed non-normal distributions across all time points. Accordingly, data are reported as medians with interquartile range (IQR). Comparisons across visits were performed using the Kruskal–Wallis test, followed by pairwise Wilcoxon (Mann–Whitney *U*) rank-sum tests with Holm–Bonferroni adjustment for *post hoc* analyses. Changes in weight and BMI between baseline and the 9-month visit were evaluated using the paired Wilcoxon signed-rank test. Missing data were handled using pairwise deletion to maximize the number of patients for each comparison.

To assess program effectiveness, two logistic regression models were fitted with binary outcome variables indicating achievement of ≥5% or ≥10% weight loss (1 = achieved; 0 = not achieved). Both models were adjusted for age, sex, initial weight, and baseline BMI category. To estimate the effect of baseline BMI category on the probability of achieving ≥5% and ≥10% weight loss, predicted values were calculated using mean age and mean initial BW for each sex.

BMI was categorized into four groups (20.0–24.9, 25.0–29.9, 30.0–34.9, and 35.0–39.9 kg/m^2^) at baseline and at program completion. A transition was defined as a change from one BMI category to another between time points. Two additional logistic regression models were fitted to assess the effects of baseline BMI, baseline BW, baseline quality of life (ORWELL-97 O × R total score), age, and sex on BMI group improvement (i.e., transition to a lower BMI category) at program completion and at the 1-year follow-up visit. The binary outcome was coded as 1 for participants whose BMI category improved and 0 otherwise.

Associations between measurements at baseline and 1-year follow-up were evaluated using Pearson’s correlation coefficients. Data cleaning was performed in Python (version 3.12.7), and statistical analyses were conducted in R (version 4.1.2). A p-value <0.05 was considered statistically significant.

## Results

### Baseline characteristics

**Figure 2:**
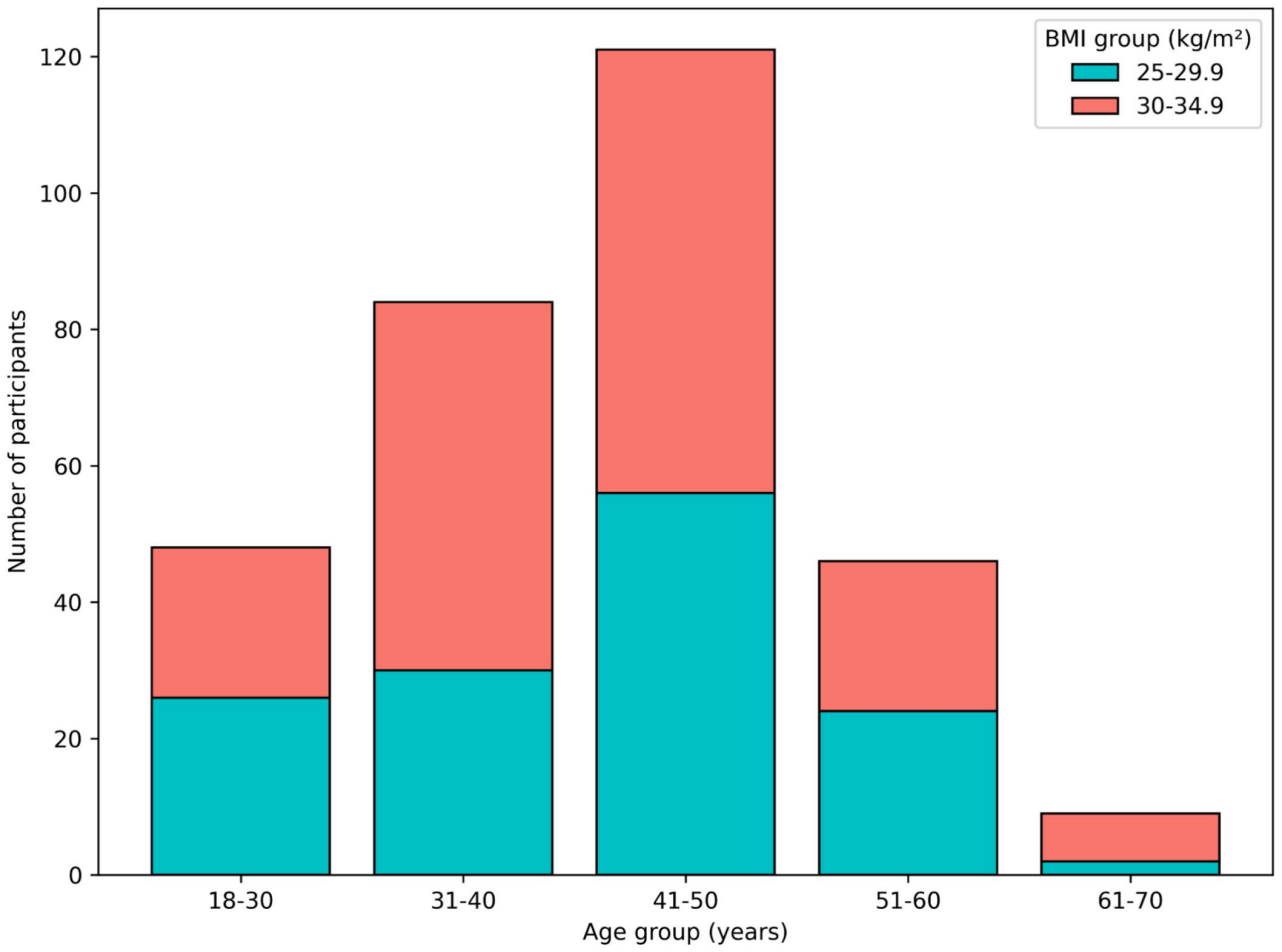
Distribution of applicants by age group and baseline BMI category.

A total of 395 individuals were initially referred to the BWMP by prevention nurses at the two sites (Registration, Figure 1), of whom 343 ultimately entered the BWMP. Complete data required for the regression analyses were available for 308 participants. Among those with disclosed demographic data, 281 were women (at baseline: age 42.7 ± 9.71 years; BW: 82.6 ± 9.15 kg; BMI 30.3 ± 2.68 kg/m^2^) and 27 were men (at baseline: age 41.7 ± 10.49 years; BW 95.5 ± 11.03 kg; BMI 31.0 ± 1.94 kg/m^2^). The distribution of participants by age and BMI categories is shown in Figure 2.

### Longitudinal changes in anthropometric, body composition, and quality-of-life measures

Table 1 presents descriptive biometric data across visits. At baseline (Visit 1, Month 0), the median BW was 83.0 kg (IQR: 76.0–90.1), with a median BMI of 30.5 kg/m^2^ (IQR: 28.1–32.3), median BFM of 31.9 kg (IQR: 28.3–37.5), and median VFA of 158.5 cm^2^ (IQR: 132.2–187.0). *p*-values for changes over time in clinical variables, body composition parameters, and ORWELL-97 scores are reported in Supplementary Table 2.

**Table 1:** Descriptive biometric and body composition data of the participants across program visits.

Parameter/time interval	0^th^ month	3^rd^ month	6^th^ month	9^th^ month	21^st^ month	33^rd^ month
BW (kg)	83 (76.0; 90.1) [*n* = 343]	79.9 (73.0; 87.0) [*n* = 274]	79 (72.5; 85) [*n* = 206]	79.3 (72.7; 85.7) [*n* = 211]	81.3 (75.6; 88.7) [*n* = 89]	79.1 (69.7; 86.7) [*n* = 15]
BMI (kg/m^2^)	30.5 (28.1; 32.3) [*n* = 343]	29.3 (27.3; 31.3) [*n* = 273]	28.7 (26.9; 30.6) [*n* = 206]	28.6 (26.9; 30.8) [*n* = 211]	29.5 (27.4; 31.5) [*n* = 87]	29.5 (26.1; 31.1) [*n* = 15]
BFM (kg)	31.9 (28.3; 37.5) [*n* = 172]	29.3 (25.2; 33.6) [*n* = 135]	28.8 (25.1; 33.3) [*n* = 104]	28.8 (23.5; 33.9) [*n* = 106]	30.6 (25.7; 33.9) [*n* = 40]	30 (24.4; 33.8) [*n* = 15]
BFP (%)	40.3 (36.8; 43.2) [*n* = 343]	38 (35; 41.2) [*n* = 271]	37.4 (33.6; 40.6) [*n* = 205]	37.3 (33.0; 41) [*n* = 210]	38.5 (35.2; 41.5) [*n* = 84]	36.8 (31.0; 41.5) [*n* = 15]
VFA (cm^2^)	158.5 (132.2; 187.0) [*n* = 172]	141.1 (117; 171.3) [*n* = 135]	138.9 (113.1; 164.3) [*n* = 104]	135.9 (109; 169.7) [*n* = 106]	146.8 (128.8; 174.1) [*n* = 40]	146.7 (110.2; 167.1) [*n* = 15]
SMM (kg)	26.6 (25.0; 29.5) [*n* = 203]	26.8 (25.0; 29.6) [*n* = 166]	27.1 (25.0; 29.8) [*n* = 122]	26.9 (24.9; 29.0) [*n* = 126]	27.1 (25.1; 30.4) [*n* = 40]	27.1 (25.4; 28.7) [*n* = 15]
InBody score	66.0 (62.0; 70.0) [*n* = 172]	69.0 (65.0; 73.5) [*n* = 135]	70.0 (65.8; 74.2) [*n* = 104]	70.0 (65; 74) [*n* = 106]	67.5 (63.0; 75.0) [*n* = 40]	69.0 (62.5; 74.5) [*n* = 15]
WC (cm)	98.0 (93.0; 104.8) [*n* = 326]	94.0 (88; 100) [*n* = 259]	94.0 (88; 98.2) [*n* = 180]	93.0 (88.0; 99.0) [*n* = 205]	96.0 (91.0; 101.5) [*n* = 83]	95.0 (85.5; 104.0) [*n* = 15]
HC (cm)	111.0 (106.0; 116.0) [*n* = 326]	109 (104.0; 113.8) [*n* = 259]	108.0 (103; 112.2) [*n* = 180]	107.0 (102.0; 112.0) [*n* = 205]	108.0 (104.0; 115.0) [*n* = 83]	110.0 (105; 112.5) [*n* = 15]
WHR	0.89 (0.85; 0.93) [*n* = 326]	0.86 (0.83; 0.91) [*n* = 259]	0.87 (0.82; 0.91) [*n* = 180]	0.87 (0.84; 0.91) [*n* = 205]	0.89 (0.85; 0.92) [*n* = 83]	0.85 (0.83; 0.90) [*n* = 15]
SBP (mmHg)	128 (118; 139) [*n* = 337]	125.5 (117; 135) [*n* = 246]	126 (118; 134) [*n* = 174]	127 (117; 137) [*n* = 209]	129 (120; 140) [*n* = 87]	130 (113; 139) [*n* = 13]
DBP (mmHg)	80 (73; 87) [*n* = 337]	79 (72; 84) [*n* = 246]	78 (73; 83) [*n* = 175]	77 (70; 83) [*n* = 208]	79 (71; 85) [*n* = 88]	77 (72; 82) [*n* = 13]
Pulse (1/min)	76 (70; 83) [*n* = 327]	74 (67; 82) [*n* = 242]	75 (68; 81) [*n* = 174]	73 (67; 82) [*n* = 208]	75 (67; 82) [*n* = 86]	68 (58; 78) [*n* = 13]
OxR	216.0 (145.5; 305.5) [*n* = 326]	NA	NA	144.0 (91.0; 218.0) [*n* = 208]	132.0 (84.0; 246.0) [*n* = 87]	112.0 (75.5; 196.5) [*n* = 15]

BFM, body fat mass; BFP, body fat percentage; BMI, body mass index; BW, body weight; DBP, diastolic blood pressure; HC, hip circumference; n, number, NA, not applicable; OxR, total score of ORWELL-97; Q1, first quartile; Q3, third quartile; SBP, systolic blood pressure; SMM, skeletal muscle mass; VFA, visceral fat area; WC, waist circumference; WHR, waist-to-hip ratio.

Significant improvements were observed in BW, BMI, and several body composition parameters—such as BFM, BFP, VFA, and InBody score—during the 3^rd^, 6^th^, and 9^th^-month visits compared to baseline. Importantly, no significant changes were noted in skeletal muscle mass despite the compulsory regular physical activity. Both WC and HC showed significant reductions at the 3^rd^, 6^th^, and 9^th^-month visits compared to baseline. The WHR demonstrated a significant improvement at the 3^rd^-month visit with no statistically significant changes at subsequent visits. No significant changes in SBP or pulse were observed across the study visits. However, DBP showed a significant reduction at the 9-month visit compared with baseline. In addition, scores on the ORWELL-97 quality-of-life questionnaire demonstrated continuous improvement throughout the study, with the 33-month results remaining superior to baseline.

### Participant flow and retention; feasibility

We defined feasibility as attendance at the 9^th^-month medical visit. Out of the 343 individuals who entered the program, only 211 attended the 9^th^-month visit (completers), resulting in a completion rate of 61.5% and a dropout rate of 38.5%. Among the 132 non-completers, 63 individuals (48%) had their last attendance at the 0^th^-month visit, 46 individuals (35%) at the 3^rd^-month visit, and 22 individuals (17%) at the 6^th^-month visit.

For the 211 individuals classified as completers, the average number of visits attended across all four time points (1^st^, 3^rd^, 6^th^, and 9^th^ month visits) was 3.86 (SD: ±0.37). Specifically, 181 individuals (85.5%) attended all four visits, 29 individuals (14%) attended three visits, and 1 individual (0.5%) attended two visits.

**Figure 3:**
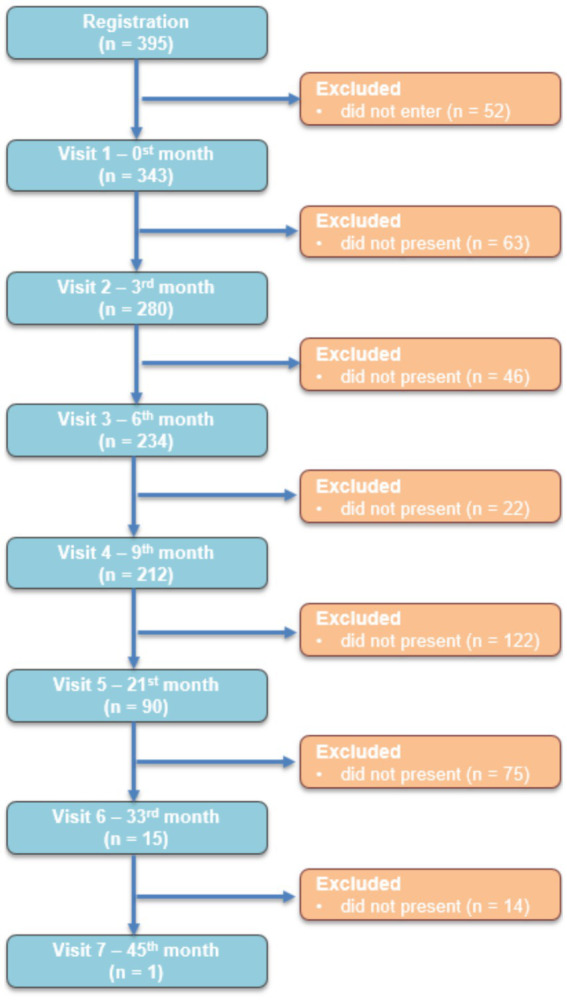
STROBE-compliant participant flow diagram across program visits.

The dropout rate observed at each assessment time point is shown in a STROBE-type flow diagram (Figure 3).

### Baseline comparison of completers and non-completers

To evaluate potential selection bias related to program completion, baseline characteristics of participants who completed the BWMP were compared with those who dropped out. Among non-completers (*n* = 98) and completers (*n* = 210), median age was 40 and 42 years, respectively (*p* = 0.053). Median baseline BMI was 30.75 kg/m^2^ in non-completers and 30.5 kg/m^2^ in completers (*p* = 0.985), while median BW was 82.65 kg and 83.35 kg, respectively (*p* = 0.952). Median baseline OxR scores were 196 among non-completers and 224 among completers (*p* = 0.604). Overall, no statistically significant differences were observed between completers and non-completers in baseline anthropometric measures or quality-of-life scores.

### Anthropometric and body composition changes; efficacy and sustainability of weight loss

The weight loss results were derived from program completers (*n* = 211). The BW of completers decreased significantly from the 1^st^ visit (month 0) (83.6 ± 9.85) to the 9^th^ month visit (79.3 ± 10.27) (*Δ* = −4.30 kg, 95% CI: −4.97 to −3.64; *p* < 0.001). A statistically significant decrease in BMI was observed from the month 0 visit to 9^th^-month visit (30.3 ± 2.57 vs. 28.8 ± 2.94) (Δ = −1.56 kg/m^2^, 95% CI: −1.81 to −1.32; *p* < 0.001), as in body fat percentage (Δ = −3.02 percentage points, 95% CI: −3.62 to −2.41; *p* < 0.001).

On an individual level, the average weight loss was 5.1 ± 5.79%, the greatest absolute reduction being 18.6 kg (−20.4%), while the largest relative loss reaching 24.5% (17.2 kg). Among the 211 completers, 19.4% (*n* = 41) lost more than 10% of their BW, 28.9% (*n* = 61) lost between 5 and 10%, and 35.1% (*n* = 74) lost less than 5%. Furthermore, 1.4% of the completers maintained their weight (*n* = 3), and 15.2% (*n* = 32) gained weight.

**Figure 4:**
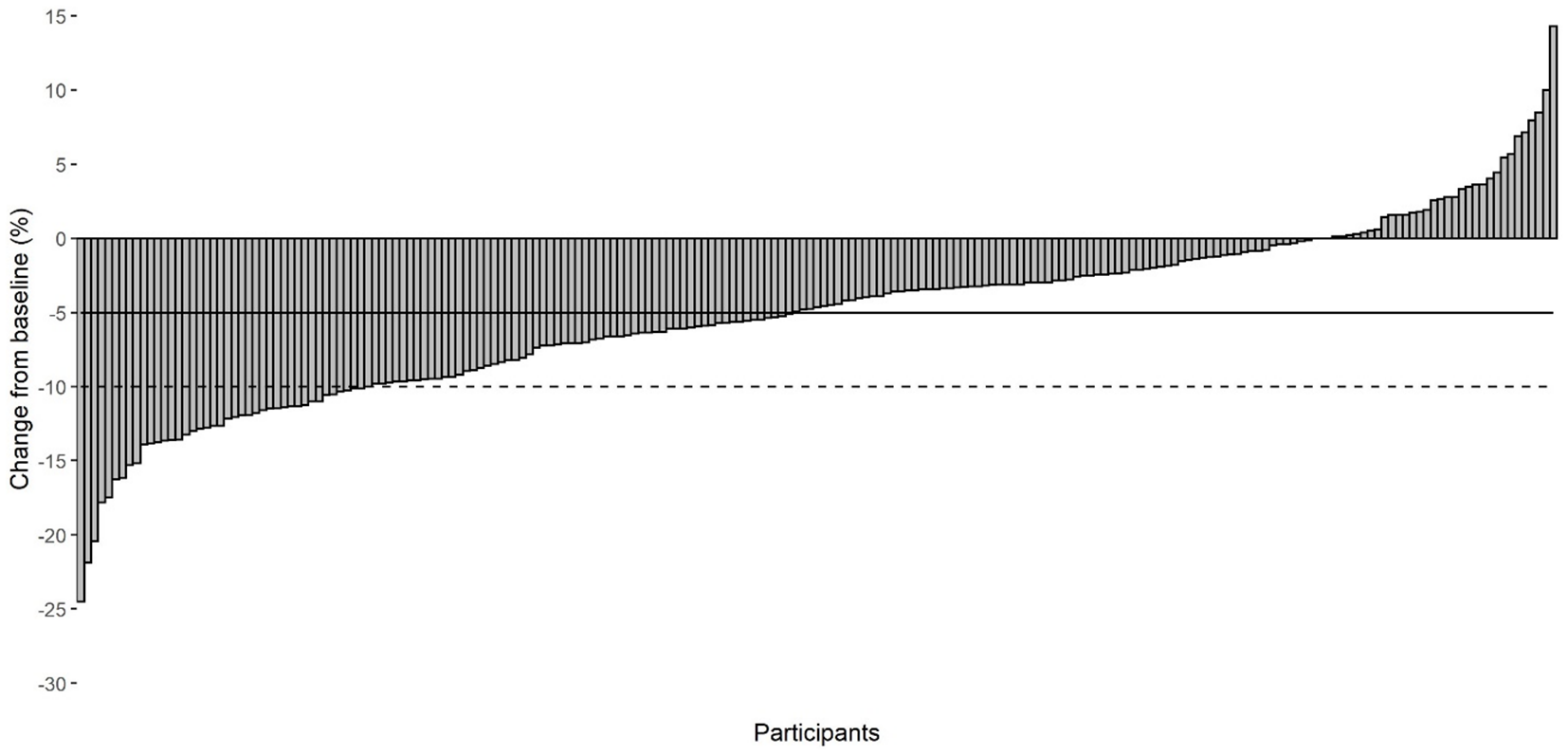
Percentage change in body weight from baseline to the final program visit for individual participants. Data are presented in a waterfall plot showing the percentage change in weight observed at the final program visit. The solid line indicates the cut-off point for patients achieving ≥5% weight loss, while the dotted line indicates the cut-off point for those achieving ≥10% weight loss.

The logistic regression model identified no increased likelihood of losing ≥5% or 10% BW when controlling for sex, age, and initial BMI (Supplementary Table 3). Based on the p-values, none of these factors were statistically significant. Although point estimates suggested a trend toward greater weight loss in participants with higher baseline BMI, this association did not reach statistical significance, likely reflecting limited statistical power. By analyzing the extent of relative weight loss using a logistic regression model (Supplementary Table 3), the results were not significant despite the apparent differences. We conducted probability predictions for achieving 5 and 10% weight loss separately for males and females, across four BMI groups. For both sex, weight and age were adjusted to their respective averages. The obtained values are shown in Supplementary Table 4 and depicted in Figure 4.

Furthermore, we analyzed how individuals from each initial BMI group transitioned by the end of the 9^th^ month, which represented in Table 2. Upon observation, it was noted that a relatively high proportion shifted to lower BMI categories, while the majority remained unchanged.

**Table 2:** The number (and percentage) of BMI group transitions among completers between baseline and the 9^th^-month visit.

BMI group (kg/m^2^) From_To	20–24.9	25–29.9	30–34.9	35–39.9	Sum
25–29.9	20 (21.5)	7 (77.5)2	1 (1)	0 (0)	93
30–34.9	0 (0)	48 (40.7)	66 (55.9)	4 (3.4)	118

Rows indicate the initial groups, columns indicate the final groups, and the numbers of individuals (also expressed as a percentage within parentheses) represent the transition to each group.

**Figure 5:**
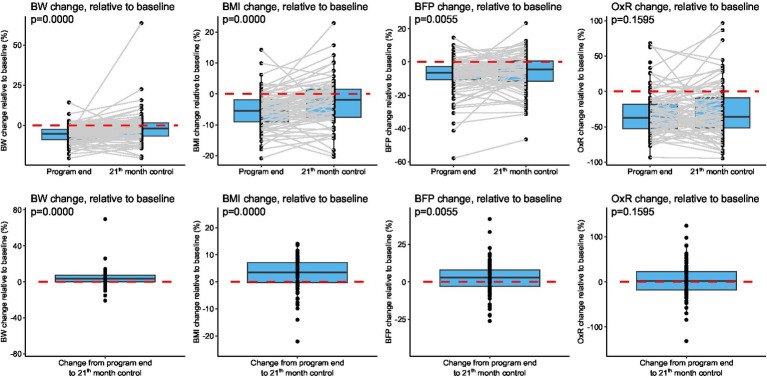
Changes in BW, BMI, BFP, and OxR score at the 1-year follow-up relative to program end. (Top) The charts indicate how the four indicators (BW, BMI, BFP, OxR) changed at the 1-year follow-up relative to the end of the program. (Bottom) The changes are perhaps more visible shown as alterations in % normalized to the end of the program. BFP, body fat percentage; BMI, body mass index; BW, body weight; OxR, total score of ORWELL-97.

The analysis of the weight loss sustainability was conducted at three time points within the completers’ population: at the month 0 (*n* = 343), at the 9^th^ month (the end of the BWMP program, *n* = 211), and at the 21^st^ month visit (the 1-year follow-up, *n* = 89). Completion of the program and return to follow-up were determined by whether BW data were available at the respective time points. Initially, we examined how four indicators (BW, BMI, BFP, OxR) changed by the end of the program and at the 1-year follow-up. Since we considered paired observations, only the 89 participants with data available for all three time points were included as shown in Figure 5. Charts were created for each characteristic. For all four characteristics, the change was normalized as a percentage by dividing the difference from the starting value by the starting value. The initial value also served as the reference for the 1-year control. Therefore, if a participant had a negative value at the end of the program and then a larger, but still negative value at the 1-year follow up, it indicates that their condition worsened since the end of the program but is still better than at the beginning. The changes are more visible as shown in Figure 5 (right) charts, where the value measured at 1 year was subtracted from the value measured at the end of the program. A positive result indicates that their condition deteriorated over the year. The four charts demonstrate that BW, BMI, and BFP significantly deteriorated by the 1-year follow-up visit.

### Quality of life outcomes

The ORWELL-97 O × R score improved significantly over the course of the intervention. Median O × R decreased from 216.0 (IQR: 145.5–305.5; *n* = 326) at baseline to 144.0 (91.0–218.0; *n* = 208) by the end of the 9-month program, indicating a substantial improvement in obesity-related quality of life. Further reductions were observed at the 21-month [132.0 (84.0–246.0); *n* = 87] and 33-month follow-ups [112.0 (75.5–196.5); *n* = 15]. Overall differences across time points were statistically significant. The reduction in O × R score from baseline to month 9 was highly significant, and the improvement was largely maintained at the 21-month follow-up, remaining significant at 33 months (*p* < 0.05). By the 45-month follow-up, changes in O × R score were no longer statistically significant (Table 1 and Supplementary Table 2).

In a completer-based analysis including participants with paired data at baseline, program completion, and 1-year follow-up (*n* = 89; Figure 5), normalized O × R changes indicated no significant deterioration in quality of life at 1 year compared with the end of the program. Although individual trajectories varied, the group-level analysis suggests that the quality-of-life improvements achieved during the BWMP were largely sustained during follow-up.

### Predictors of weight loss and BMI category change

Given that a relatively large number of participants were able to shift to a lower BMI category during the BWMP, we conducted a logistic regression model to determine which baseline characteristic can predict a change of BMI category by the end of the program (e.g., from 25 to 29.9 kg/m^2^ to 20–24.9 kg/m^2^, or from 30 to 35 kg/m^2^ to one of the lower categories).

**Figure 6:**
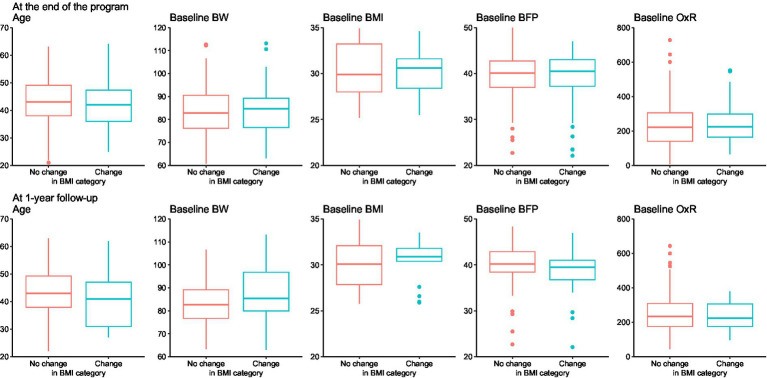
Distribution of selected baseline characteristics according to BMI category change status at program end (top) and at the 1-year follow-up (bottom).

In the regression model (Table 3), the initial BW, initial BMI category, initial BFP, initial OxR value, age, and gender were included as predictors. According to the model, only the initial BMI had a significant influence, with an odds ratio of 0.8, indicating that the probability of transitioning to a lower BMI category decreases as the initial BMI increases. Visual presentation of measured parameters according to shifts to lower BMI category are shown on Figure 6.

**Table 3:** Logistic regression model examining the association between baseline characteristics and BMI category change by program end.

Predictor variable	Odds ratio (95% CI)	*P*-value
Age (year)	0.99 (0.96–1.03)	n.s.
Female gender	1.10 (0.28–4.38)	n.s.
Initial BW (kg)	1.05 (1.00–1.10)	n.s.
Initial BMI (kg/m^2^)	0.80 (0.66–0.99)	***<0.05***
Initial BFP (%)	1.03 (0.95–1.11)	n.s.
Initial OxR	1.00 (0.998–1.003)	n.s.

BFP, body fat percentage; BMI, body mass index; BW, body weight; OxR, total score of ORWELL-97; CI, confidence interval; n.s., not significant. The italicized bold value in Table 3 indicate statistically significant *p*-value.

Another regression model (Supplementary Table 5) was constructed to determine what influenced the likelihood of “BMI category decrease” at the 1-year follow-up compared to the initial measurement (among those individuals who returned). The same set of predictors as the previous model were used, with the addition of the BMI achieved at the end of the program. According to this model, the magnitude of the BMI achieved at the end of the program had the greatest influence on the decrease in category achieved at the 1-year follow-up, although this influence was not statistically significant.

We checked whether BMI category change at the end of the BWMP was associated with other characteristics. We found no significant difference when comparing age, BW, BMI, BFP and OxR between participants with and without BMI category change. The same was observed for the 1-year follow-up.

### Correlations

**Figure 7:**
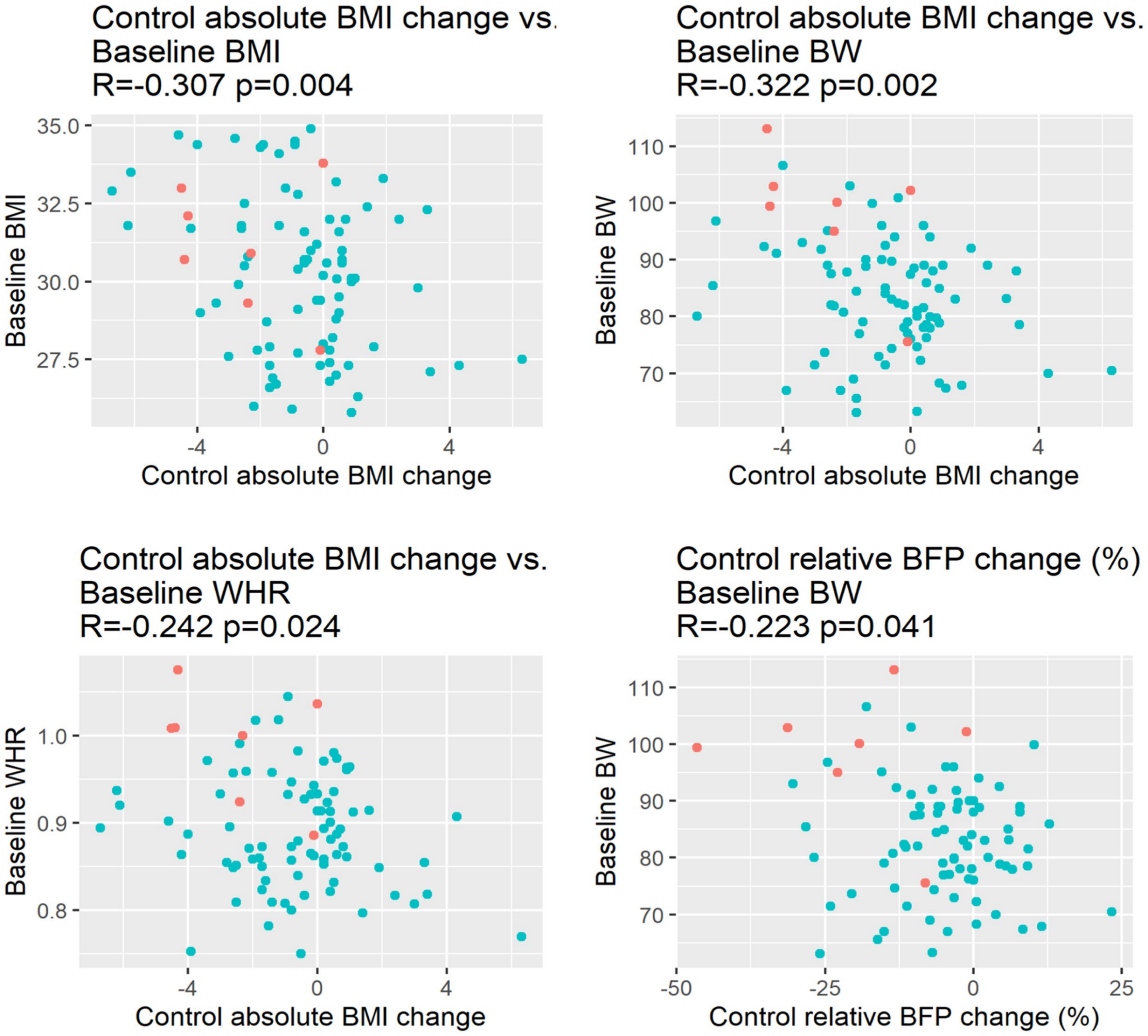
Correlations between BMI change at program end (at the 9^th^-month visit) and baseline BMI, BW, and WHR. BFP, Body fat percentage; BMI, body mass index; BW, body weight; WHR, waist-to-hip ratio.

The BMI change, measured at the end of the program (at the 9^th^-month visit), correlated weakly, but significantly with the initial BMI, initial BW, and initial waist-hip ratio as shown on Figure 7.

## Discussion

### Design of the BWMP

The BWMP was a relatively large-scale interventional program for adults living with overweight and mild obesity in Hungary, conducted in a small community in the southeastern part of the country, an area heavily affected by the burden of obesity. A multidisciplinary team – including obesity specialist, dieticians, psychologists, trainers, prevention nurses, and physical activity advisors – provided guidance and support to participants committed to reducing their BW, improving body composition, and enhancing fitness through regular medical and dietary consultations and structured physical activity. Ongoing monitoring was essential to the success of the program.

The BWMP was developed in alignment with international standards for the management of obesity, with the aim of delivering a streamlined yet comprehensive set of multidisciplinary interventions tailored to the needs of Hungarian patients (18, 27, 28).

To prevent weight regain, the clinical guidelines from the USA, Canada, and Europe recommend participation in structured weight-loss maintenance programs at least 1 year, with counseling provided at least monthly. In the present study, participants received follow-up care for a total duration of 2 years.

When positioning the BWMP in the context of landmark international obesity and diabetes prevention trials, several key interventional programs must be highlighted. The DPP (19) in the United States demonstrated that intensive lifestyle intervention targeting modest weight loss (7% of initial BW) and increased physical activity (150 min/week) reduced the incidence of type 2 diabetes by 58% compared with placebo. The Look AHEAD (20) study, a large randomized controlled trial among individuals with type 2 diabetes, confirmed that intensive lifestyle intervention led to significant and sustained weight loss (8.6% at 1 year, 6% at 8 years). Participants in the intensive lifestyle intervention reported greater improvements in physical functioning, general health, and vitality scores compared with the diabetes support and education group, reflecting an overall enhancement in health-related quality of life. Similarly, the Da Qing (21) in China was the first to show that lifestyle intervention among individuals with impaired glucose tolerance reduced the risk of developing diabetes by 31–46% over a 6-year period and delayed the onset of diabetes for up to 14 years after the active intervention. However, whether such lifestyle interventions also reduce cardiovascular disease and mortality remained unclear at that time (21, 29). More recently, the DIRECT trial (22) in the UK demonstrated that intensive primary care–led weight management achieved diabetes remission in nearly half of participants at 12 months, with sustained benefits at 24 months. These landmark interventions have established the foundation for modern obesity and diabetes prevention strategies and underscore the critical role of structured, multidisciplinary lifestyle programs.

The primary objective of the BWMP was to halt further weight gain among participants, thereby preventing progression to more severe obesity and its potential long-term consequences, while also encouraging participants to educate their families and communities and promote healthier lifestyles, contributing to the development of a more health-conscious future generation. The program was not designed to demonstrate delayed onset of diabetes or remission of prediabetes, but rather to foster a healthier and more physically fit population. When considered in the context of the major international prevention trials cited above, it may be concluded that any intervention aimed at improving overall health status and body composition has the potential to delay the development of metabolic and other complications, thereby reducing both individual and societal healthcare burdens.

### Descriptive biometric data and clinical variables of the participants

Individuals enrolled in the BWMP at two sites were predominantly female. A similarly high trend in female participation rates for weight loss programs can be also observed in the literature, ranging from 63 to 86% (30–33). Based on our observations, male participants often joined the program due to the influence of their female partners who were also part of the study. Further sociodemographic and psychological research/analysis would be necessary to clarify the reasons behind the disproportionate female participation.

Our structured educational program was most effective for weight loss during the first 3 months. Significant improvements in BW, BMI, and specific InBody parameters—including body fat mass, BFP, VFA, and InBody score—were observed at the 3^rd^, 6^th^, and 9^th^-month visits when compared to baseline. Increased VFA was found to be as an independent predictor for cardiovascular events, cardiovascular death, and all-cause death (24). In addition to smoking, sex, and BMI, visceral fat area was found to significantly impact carotid intima-media thickness, a marker of the preclinical stage of atherosclerosis, in the Chinese population with type 2 diabetes (34). Consistent with the improvements in VFA, both waist and hip circumferences showed significant reductions at the 3^rd^, 6^th^, and 9^th^-month visits, further reinforcing the reliability and relevance of these easily accessible, classic biometric parameters. Importantly, no significant changes were noted in skeletal muscle mass, indicating that the applied comprehensive BWMP effectively prevented the loss of skeletal muscle mass. While changes in clinical parameters were no longer significant at 21, 33, and 45 months compared to baseline, the improvement in quality of life persisted. Notably, participants experienced enhanced quality of life after completing the program, regardless of BMI or body composition changes. No significant changes in SBP or pulse were detected across the visits; however, a significant decrease in DBP was observed at the 9^th^-month visit compared to baseline. In a study involving 56 patients with metabolic syndrome, a 3-month physical training period was deemed effective, as evidenced by a significant decrease in BMI, along with a significant reduction in both systolic and diastolic blood pressure (35), underlying our results. It is important to note that the current study was preventive and health-maintenance in nature. Although there were hypertensive and hypothyroid patients among the participants, they were stable and well-controlled with medication.

### Feasibility, dropout and retention rates of the BWMP

Retention and dropout rates vary wildly across lifestyle-intervention studies, with follow-up typically extending up to a maximum of 12 months (30, 36, 37), most commonly limited to approximately 6 months after program initiation (31, 32, 38). Although the active phase in our study lasted 9 months, the 1 and 2 year follow-up visits provide valuable long-term data, yielding important insights beyond the intervention period. The majority of published studies report outcomes at 6 months, we used this time point for comparison. As shown in Figure 3 out of the 343 participants who were initially enrolled in the BWMP, 234 attended the 6^th^-month visit, corresponding to a dropout rate of 31.8% and a retention rate of 68.2%. This compares with the study by Trepanowski et al. (32), in which the dropout rates ranged from 29 to 38% across different intervention groups. Our retention rate was also higher than those by Perna et al. (30), (44.4% dropout rate) and Colombo et al. (31) (57% dropout rate). Moreover, compared with the 22% retention reported by Finley et al. (37), and the 65% by Vázquez-Velázquez and García García (33), the 68.2% retention observed in our cohort appears favorable. However, in our study feasibility was defined as attendance at the 9^th^-month visit, yielding a completion rate of 61.5%. Because most studies report outcomes at 6 and 12 months after initiation, direct comparison with our results is limited. Among the 211 completers, the mean number of visits attended across the four scheduled time points (1^st^, 3^rd^, 6^th^, and 9^th^ months) was 3.86 ± 0.37. Notably, 85.5% attended all four visits, 14% attended three, and 0.5% attended only two (33).

### Effectiveness and weight loss sustainability of the BWMP

The weight loss results were based on individuals who completed the program (*n* = 211). The weight (*Δ* = −4.30 kg) and, correspondingly, the BMI (*Δ* = −1.56 kg/m^2^) of the completers showed a significant decrease from the first visit (month 0) to the 9^th^-month visit.

The logistic regression analysis indicated that, after adjusting for sex, age, and baseline BMI, neither ≥5% nor 10% BW loss was significantly more likely (Table 3). However, other factors did not demonstrate predictive significance. Nevertheless, several studies have shown that higher age is associated with greater program completion and greater weight loss (33, 39, 40). However, in our cohort, there was no meaningful difference in age between participants who completed the program and those who dropped out. Table 3 shows that by the end of the 9^th^ month, a relatively high proportion of individuals moved to lower BMI categories, though most remained in their initial category.

The mean weight loss was 5.1%. The greatest absolute weight loss was 18.6 kg (20.4%), while the largest relative weight reduction was 24.5% (17.2 kg). Among the 211 BWMP completers, 19.4% achieved a weight loss more than 10% of their initial BW, 28.9% lost between 5 and 10%, and 35.1% lost less than 5%. In addition, 1.4% of participants maintained their weight, while 15.2% experienced weight gain. Because our intervention lasted 9 months – longer than the typical 6-month short-term programs – direct comparison with many published studies is limited, as these often differ in duration, methodology, and patient populations. For example, in the study by Vázquez-Velázquez and García García (33), 40% of participants completing the comprehensive obesity care program at a public tertiary hospital achieved clinically meaningful weight loss (≥5%) within 6 months. In contrast, in our ambulatory, prevention-oriented program, 48.3% of completers achieved at least 5% weight loss over the 9-month intervention period.

In the study by Buscemi et al. (41) the scores of the ORWELL 97 questionnaire – measuring the quality of life of people living with obesity – were significantly correlated with the percentage of BW change 10 years after medical nutritional treatment (41). In our cohort weight loss sustainability was evaluated at three time points: month 0 (*n* = 343), month 9 (end of the program, *n* = 211), and 1-year follow-u (*n* = 89). Changes in BW, BMI, BFP, and OxR were analyzed between month 9 and the 1-year follow-up (Figure 6). Increases in relative BW, BMI, and BFP indicated partial deterioration of anthropometric outcomes. In contrast, the OxR scores remained stable, suggesting that the improvements in obesity-related quality of life achieved by the end of the program were sustained at the 1-year follow-up. This represents one of the key findings of our present study.

Several limitations should be considered when interpreting the findings of this study. First, the non-randomized study design and the absence of a control group limit causal inference and restrict direct comparison with controlled interventional trials. Although data were collected prospectively, the analysis was retrospective and embedded within a real-world public health program. Second, no formal sample size calculation was performed, which may have limited the statistical power of the study. Third, loss to follow-up and potential attrition bias may have influenced the observed outcomes, as not all participants completed the active program or attended all follow-up visits. This attrition, together with the fact that the analyzed cohort consisted predominantly of women, may further limit the generalizability of the findings. Fourth, heterogeneity in body composition assessment represents an additional limitation that may have affected data consistency. During the first 3 years of the program, bioimpedance measurements were obtained using different devices because the InBody analyzer was not initially available for financial reasons. Consequently, measurements were derived from two different methodologies, resulting in limited overlap between datasets across time points. Although sensitivity analyses restricted to InBody 770 measurements yielded consistent results, this technical heterogeneity may still have introduced measurement variability. Fifth, although certain biochemical and socioeconomic parameters were collected, these data were not included in the present analysis. Sixth, the limited power of the regression models restricts the interpretation of predictors of weight loss and BMI category changes. Several observed trends, such as the apparent association between higher baseline BMI and greater weight loss, did not reach statistical significance, likely reflecting limited statistical power. Finally, although the BWMP shares conceptual elements with landmark randomized controlled lifestyle intervention trials —such as the DPP (19), Look AHEAD (20), Da Qing (21, 29), and DIRECT (22) — it differs fundamentally in its pragmatic, community-based, non-randomized implementation. These trials demonstrated the efficacy of structured lifestyle interventions under controlled conditions, whereas the BWMP evaluates their translation into routine public healthcare practice. Accordingly, while direct comparison is not appropriate, the BWMP provides complementary insights on feasibility, scalability, and sustainability of a complex behavioral intervention in a real-world setting. Weight loss among completers was modest and tended to attenuate at 1 year follow-up, whereas improvements in obesity-related quality of life appeared more stable in those returning for follow-up; however, these findings, cannot be generalized to all participants. Moreover, the study population consisted of motivated individuals without diabetes or severe comorbidities, which further limits the direct comparison of results with data from studies conducted in diabetic an prediabetic patients, which further limit the generalizability of our results to those settings.

## Conclusion

The BWMP represents a feasible and acceptable multidisciplinary intervention for a subset of adults living with overweight and mild obesity in southern Hungary who were motivated to improve their health and physical fitness. The program integrated medical, dietary, psychological, and physical activity support delivered by a multidisciplinary team and was accompanied by regular monitoring of behavioral change. Although the greatest weight reduction occurred during the first 3 months, participants who completed the active phase of the program experienced significant improvements in obesity-related quality of life, which were maintained during follow-up. These findings suggest that a structured, community-based, multidisciplinary approach may be a viable model for delivering obesity care within the Hungarian public healthcare system, and that frequent patient contact and education can support clinically meaningful weight loss in a proportion of participants.

## Data Availability

The original contributions presented in the study are included in the article/Supplementary material, further inquiries can be directed to the corresponding author.
