# Supplementary material for: Weight, body composition and quality of life changes in a Hungarian community-based body weight management program: an observational cohort analysis
Source: Front Public Health. 2026 Feb 19;14:1751402. doi: 10.3389/fpubh.2026.1751402 (PMC12961615; doi:10.3389/fpubh.2026.1751402)
Supplement: Supplementary file 1 [file Data_Sheet_1.docx]

**SUPPLEMENTARY MATERIAL**

***Research Article***

***Weight, body composition and quality of life changes in a Hungarian community-based body weight management program: an observational cohort analysis***

Éva Csajbók^1, 2*^, Sándor Bordé^3^, Terézia Páhi^1^, Anita Kollárné Korsós^1^, Zsuzsanna Gyurisné Pethő^1^, Anna Vágvölgyi^2†^, Árpád Kallai^1†^

**Supplementary Table 1.** STROBE checklist for cohort studies.

| **Section** | **Item No.** | **STROBE recommendation** | **Addressed in manuscript** |
| --- | --- | --- | --- |
| **Title and abstract** | **1a** | Indicate the study’s design with a commonly used term in the title or abstract | Title; Abstract (Methods: “non-randomized, prospective observational study”) |
|  | **1b** | Provide an informative and balanced summary of what was done and what was found | Abstract |
| **Introduction** | **2** | Explain the scientific background and rationale | Introduction |
|  | **3** | State specific objectives, including prespecified hypotheses | Introduction (Objectives stated; exploratory observational design) |
| **Methods** | **4** | Present key elements of study design early in the paper | Methods – Study design and setting of BWMP |
|  | **5** | Describe the setting, locations, and relevant dates | Methods – Study design and setting of BWMP; The aim and structure of the BWMP, recruitment data |
|  | **6a** | Give eligibility criteria and methods of participant selection and follow-up | Methods – Inclusion and exclusion criteria; Medical consultation, clinical variables, body composition; Dietary intervention; Physical activity; Psychological support |
|  | **6b** | For matched studies, give matching criteria | Not applicable (non-matched observational cohort) |
|  | **7** | Clearly define outcomes, exposures, predictors, confounders | Methods – Clinical variables, body composition, Statistical analysis |
|  | **8** | Describe data sources and measurement methods | Methods – Medical consultation, body composition, ORWELL-97 questionnaire |
|  | **9** | Describe efforts to address potential sources of bias | Methods – Statistical analysis; Discussion |
|  | **10** | Explain how study size was determined | Methods – Real-world community program; no a priori sample size calculation |
|  | **11** | Explain handling of quantitative variables | Methods – Statistical analysis |
|  | **12a** | Describe statistical methods used to control for confounding | Methods – Logistic regression models |
|  | **12b** | Describe methods for subgroup and interaction analyses | Methods – Statistical analysis |
|  | **12c** | Explain how missing data were addressed | Methods – Statistical analysis (pairwise deletion) |
|  | **12d** | Explain how loss to follow-up was addressed | Results – Participant flow; Figure 3 |
|  | **12e** | Describe sensitivity analyses | Methods – Body composition analysis: device sensitivity analysis |
| **Results** | **13a** | Report numbers of individuals at each study stage | Results – Participant flow; Figure 3 |
|  | **13b** | Give reasons for non-participation | Results – Feasibility and dropout |
|  | **13c** | Consider use of a flow diagram | Figure 3 |
|  | **14a** | Give characteristics of study participants | Results – Baseline characteristics |
|  | **14b** | Indicate number of participants with missing data | Table 1; Results |
|  | **14c** | Summarize follow-up time | Results – Follow-up duration and longitudinal outcome assessment (3, 6, 9, 21, and 33 months) |
|  | **15** | Report outcome events or summary measures over time | Results – Tables 1–3; Figures 4–7 |
|  | **16a** | Give unadjusted and adjusted estimates with precision | Results – Regression models; confidence intervals |
|  | **16b** | Report category boundaries when continuous variables categorized | Methods – BMI categories |
|  | **16c** | Translate relative risk into absolute risk where relevant | Not applicable |
|  | **17** | Report other analyses (subgroups, sensitivity) | Results; Supplementary Tables |
| **Discussion** | **18** | Summarize key results with reference to objectives | Discussion |
|  | **19** | Discuss limitations, including bias and imprecision | Discussion |
|  | **20** | Provide cautious overall interpretation | Discussion |
|  | **21** | Discuss generalisability (external validity) | Discussion |
| **Other information** | **22** | Give funding source and role of funders | Funding section |

**Supplementary Table 2**. Clinical variables, InBody parameters, and the total score of ORWELL-97 of the participants compared across visits using Kruskal-Wallis test, followed by a pairwise Wilcoxon rank-sum test with Holm-Bonferroni adjustment.

| **P-value** | **0^th^ month** | **3^rd^ month** | **6^th^ month** | **9^th^ month** | **21^st^ month** |
| --- | --- | --- | --- | --- | --- |
| **BW (kg)** Kruskal-Wallis p=0.000 |  | | | | |
| 3^rd^ month | ***< 0.01*** | NA | NA | NA | NA |
| 6^th^ month | ***< 0.001*** | n.s. | NA | NA | NA |
| 9^th^ month | ***< 0.001*** | n.s. | n.s. | NA | NA |
| 21^st^ month | n.s. | n.s. | n.s. | n.s. | NA |
| 33^rd^ month | n.s. | n.s. | n.s. | n.s. | n.s. |
| **BMI (kg/m^2^)** Kruskal-Wallis p=0.000 |  | | | | |
| 3^rd^ month | ***< 0.001*** | NA | NA | NA | NA |
| 6^th^ month | ***< 0.0001*** | n.s. | NA | NA | NA |
| 9^th^ month | ***< 0.0001*** | n.s. | n.s. | NA | NA |
| 21^st^ month | n.s. | n.s. | n.s. | n.s. | NA |
| 33^rd^ month | n.s. | n.s. | n.s. | n.s. | n.s. |
| **BFM (kg)** Kruskal-Wallis p=0.000 |  | | | | |
| 3^rd^ month | ***< 0.01*** | NA | NA | NA | NA |
| 6^th^ month | ***< 0.001*** | n.s. | NA | NA | NA |
| 9^th^ month | ***< 0.001*** | n.s. | n.s. | NA | NA |
| 21^st^ month | n.s. | n.s. | n.s. | n.s. | NA |
| 33^rd^ month | n.s. | n.s. | n.s. | n.s. | n.s. |
| **BFP (%)** Kruskal-Wallis p=0.000 |  | | | | |
| 3^rd^ month | ***< 0.001*** | NA | NA | NA | NA |
| 6^th^ month | ***< 0.001*** | n.s. | NA | NA | NA |
| 9^th^ month | ***< 0.001*** | n.s. | n.s. | NA | NA |
| 21^st^ month | n.s. | n.s. | n.s. | n.s. | NA |
| 33^rd^ month | n.s. | n.s. | n.s. | n.s. | n.s. |
| **VFA (cm^2^)** Kruskal-Wallis p=0.000 |  | | | | |
| 3^rd^ month | ***< 0.001*** | NA | NA | NA | NA |
| 6^th^ month | ***< 0.001*** | n.s. | NA | NA | NA |
| 9^th^ month | ***< 0.001*** | n.s. | n.s. | NA | NA |
| 21^st^ month | n.s. | n.s. | n.s. | n.s. | NA |
| 33^rd^ month | n.s. | n.s. | n.s. | n.s. | n.s. |
| **SMM (kg)** Kruskal-Wallis p=0.975 |  | | | | |
| 3^rd^ month | n.s. | NA | NA | NA | NA |
| 6^th^ month | n.s. | n.s. | NA | NA | NA |
| 9^th^ month | n.s. | n.s. | n.s. | NA | NA |
| 21^st^ month | n.s. | n.s. | n.s. | n.s. | NA |
| 33^rd^ month | n.s. | n.s. | n.s. | n.s. | n.s. |
| **IBS** Kruskal-Wallis p=0.000 |  |  |  |  |  |
| 3^rd^ month | ***< 0.001*** | NA | NA | NA | NA |
| 6^th^ month | ***< 0.001*** | n.s. | NA | NA | NA |
| 9^th^ month | ***< 0.001*** | n.s. | n.s. | NA | NA |
| 21^st^ month | n.s. | n.s. | n.s. | n.s. | NA |
| 33^rd^ month | n.s. | n.s. | n.s. | n.s. | n.s. |
| **WC (cm)** Kruskal-Wallis p=0.000 |  |  |  |  |  |
| 3^rd^ month | ***< 0.0001*** | NA | NA | NA | NA |
| 6^th^ month | ***< 0.0001*** | n.s. | NA | NA | NA |
| 9^th^ month | ***< 0.0001*** | n.s. | n.s. | NA | NA |
| 21^st^ month | n.s. | n.s. | n.s. | n.s. | NA |
| 33^rd^ month | n.s. | n.s. | n.s. | n.s. | n.s. |
| **HC (cm)** Kruskal-Wallis p=0.000 |  | | | | |
| 3^rd^ month | ***< 0.01*** | NA | NA | NA | NA |
| 6^th^ month | ***< 0.001*** | n.s. | NA | NA | NA |
| 9^th^ month | ***< 0.0001*** | n.s. | n.s. | NA | NA |
| 21^st^ month | n.s. | n.s. | n.s. | n.s. | NA |
| 33^rd^ month | n.s. | n.s. | n.s. | n.s. | n.s. |
| **WHR** Kruskal-Wallis p=0.015 |  | | | | |
| 3^rd^ month | ***< 0.05*** | NA | NA | NA | NA |
| 6^th^ month | n.s. | n.s. | NA | NA | NA |
| 9^th^ month | n.s. | n.s. | n.s. | NA | NA |
| 21^st^ month | n.s. | n.s. | n.s. | n.s. | NA |
| 33^rd^ month | n.s. | n.s. | n.s. | n.s. | n.s. |
| **SBP (mmHg)** Kruskal-Wallis p=0.155 |  | | | | |
| 3^rd^ month | n.s. | NA | NA | NA | NA |
| 6^th^ month | n.s. | n.s. | n.s. | NA | NA |
| 9^th^ month | n.s. | n.s. | n.s. | NA | NA |
| 21^st^ month | n.s. | n.s. | n.s. | n.s. | NA |
| 33^rd^ month | n.s. | n.s. | n.s. | n.s. | n.s. |
| **DBP (mmHg)** Kruskal-Wallis p=0.012 |  | | | | |
| 3^rd^ month | n.s. | NA | NA | NA | NA |
| 6^th^ month | n.s. | n.s. | NA | NA | NA |
| 9^th^ month | ***< 0.01*** | n.s. | n.s. | NA | NA |
| 21^st^ month | n.s. | n.s. | n.s. | n.s. | NA |
| 33^rd^ month | n.s. | n.s. | n.s. | n.s. | n.s. |
| **Pulse (1/min)** Kruskal-Wallis p=0.017 |  | | | | |
| 3^rd^ month | n.s. | NA | NA | NA | NA |
| 6^th^ month | n.s. | n.s. | NA | NA | NA |
| 9^th^ month | n.s. | n.s. | n.s. | NA | NA |
| 21^st^ month | n.s. | n.s. | n.s. | n.s. | NA |
| 33^rd^ month | n.s. | n.s. | n.s. | n.s. | n.s. |
| **OxR** Kruskal-Wallis p=0.000 |  | | | | |
| 9^th^ month | ***< 0.0001*** | -- | -- | NA | NA |
| 21^st^ month | ***< 0.0001*** | -- | -- | n.s. | NA |
| 33^rd^ month | ***< 0.05*** | -- | -- | n.s. | n.s. |

*BFM, body fat mass; BFP, body fat percentage; BMI, body mass index; BW, body weight; DBP, diastolic blood pressure; HC, hip circumference; n, number, NA, not applicable; n.s.: not significant; OxR, total score of ORWELL-97; SBP, systolic blood pressure; SMM, skeletal muscle mass; VFA, visceral fat area; WC, waist circumference; WHR, waist-to-hip ratio.*

**Supplementary Table 3.** Analyzing the extent of relative weight loss using a logistic regression model.

|  | **Odds Ratio** | | **Confidence Interval 95%** | | | | **P-value** | |
| --- | --- | --- | --- | --- | --- | --- | --- | --- |
|  | **5%** | **10%** | **5%** | | **10%** | | **5%** | **10%** |
|  |  | | **Lower** | **Upper** | **Lower** | **Upper** |  | |
| **Age (years)** | 1.01 | 0.99 | 0.98 | 1.04 | 0.95 | 1.03 | n.s.. | n.s.. |
| **Baseline weight (kg)** | 0.99 | 1.01 | 0.95 | 1.03 | 0.96 | 1.06 | n.s.. | n.s.. |
| **Female gender** | 1.31 | 1.19 | 0.41 | 4.24 | 0.28 | 5.142 | n.s.. | n.s.. |
| **Initial BMI: 30-34.9 kg/m^2^** | 1.28 | 1.61 | 0.61 | 2.69 | 0.62 | 4.15 | n.s.. | n.s.. |

*n.s..: not significant.*

**Supplementary Table 4.** Probability prediction for achieving 5% and 10% weight loss, separately for males and females, across four BMI groups as starting values. For each gender, weight and age were adjusted to their respective averages.

| **Gender** | **Weight (kg)** | **Age (year)** | **BMI group (kg/m^2^)** | **Probability** | |
| --- | --- | --- | --- | --- | --- |
|  |  |  |  | **Min. 5%** | **Min. 10%** |
| **Male** | **97.2** | **41.3** | **25-29.9** | 0.353 | 0.144 |
|  |  |  | **30-34.9** | 0.412 | 0.212 |
| **Female** | **82.6** | **42.6** | **25-29.9** | 0.459 | 0.155 |
|  |  |  | **30-34.9** | 0.521 | 0.227 |

**Supplementary Table 5**. Logistic regression model determining the parameters influencing the likelihood of BMI category decrease at the 1-year follow-up compared to the initial measurement. BFP, body fat percentage; BMI, body mass index; BW, body weight; OxR, total score of ORWELL-97; CI, confidence interval; n.s., not significant.

|  | **Odds Ratio (95% CI)** | **P-value** |
| --- | --- | --- |
| **Age (years)** | 0.98 (0.92–1.04) | n.s. |
| **Female gender** | 0.20 (0.02–2.47) | n.s. |
| **Initial BW (kg)** | 1.01 (0.92–1.12) | n.s. |
| **Initial BMI (kg/m^2^)** | 1.30 (0.78–2.16) | n.s. |
| **Final BMI (kg/m^2^)** | 0.70 (0.48–1.03) | n.s. |
| **Initial BFP (%)** | 1.00 (0.86–1.17) | n.s. |
| **Initial OxR** | 1.00 (0.995–1.005) | n.s. |
